# Transcriptomic Landscape of Gut Microbiota–Host Interactions Reveals Domestication-Related Changes in the Pearl Oyster *Pinctada maxima*

**DOI:** 10.3390/ani16182849

**Published:** 2026-09-10

**Authors:** Jing Huang, Teng Zhang, Dongmei Xie, Zhe Zheng, Chuangye Yang, Yongshan Liao, Qingheng Wang, Yuewen Deng

**Affiliations:** 1 Shenzhen Institute, Guangdong Ocean University, Shenzhen 518120, China; huangjing81@stu.gdou.edu.cn (J.H.); 19727507350@163.com (D.X.); zhengzhe@gdou.edu.cn (Z.Z.); 2 Fisheries College, Guangdong Ocean University, Zhanjiang 524088, China; 2112201018@stu.gdou.edu.cn (T.Z.); wangqh@gdou.edu.cn (Q.W.); 3 Pearl Research Institute, Guangdong Ocean University, Zhanjiang 524088, China; liaoyongshan@gdou.edu.cn; 4 Zhanjiang Engineering Research Center of Marine Pearl Cultivation and High-Value Utilization, Guangdong Zunding Pearl Co., Ltd., Zhanjiang 524088, China; 5 Pearl Breeding and Processing Engineering Technology Research Centre of Guangdong Province, Zhanjiang 524088, China; 6 Guangdong Provincial Key Laboratory of Aquatic Animal Disease Control and Healthy Culture, Zhanjiang 524088, China

**Keywords:** *Pinctada maxima*, domesticated population, adaptation, transcriptome, gut microbiota

## Abstract

The offspring of domesticated *Pinctada maxima* exhibited various physiological and microbial adjustments to the complex and dynamic conditions of coastal environments. To support the restoration of *P. maxima* genetic resources and explore the molecular mechanisms underlying these phenotypic responses, we conducted a comparative analysis of the intestinal transcriptome and microbiota of wild parental and domesticated generations. Each sample generated an average of 43,600,525 clean reads, which were mapped to the *P. maxima* reference genome with mapping rates ranging from 63.91% to 79.48%, and a total of 3007 differentially expressed genes (DEGs) were subsequently identified. Gene Ontology analysis revealed that the DEGs were enriched in organic acid metabolism, and Kyoto Encyclopedia of Genes and Genomes (KEGG) analysis revealed that the DEGs were enriched in glycosphingolipid biosynthesis and xenobiotic metabolism via cytochrome P450. Microbiota profiling revealed significant compositional shifts at phylum and genus levels, with increased alpha diversity in F1; dominant phyla transitioned towards Spirochaetota and Bacteroidota, and functional predictions pointed to enhanced metabolic and immune capacities. Quantitative validated the up-regulation of immune genes (*PmHR96h*, *PmIAP*) and down-regulation of calcium-signaling genes (*PmCaM*, *PmHSP90*), consistent with RNA-seq data. Collectively, these coordinated transcriptomic and microbial alterations reflect a multifaceted host–microbiome adaptive response to nearshore conditions. Our findings provide valuable molecular markers and microbial indicators for selective breeding and health monitoring, offering a scientific basis for improving the resilience of *P. maxima* aquaculture under changing environmental conditions.

## Simple Summary

Wild stocks of the pearl oyster *Pinctada maxima* have declined, prompting domestication for aquaculture, yet juvenile *P. maxima* farm mortality remains high and poorly understood. Comparing wild parents with first-generation domesticated offspring, the present study found that domesticated *P. maxima* upregulated gut genes for immunity, vitamin uptake, and energy metabolism, downregulated neural pathways, and shifted gut microbiota toward beneficial bacteria. These changes suggest active physiological adaptation to farm conditions. Results offer correlative molecular and microbial evidence for domestication-related adjustments, and identify candidate biomarkers for future breeding, though production traits were not directly assayed.

## 1. Introduction

*Pinctada maxima*, a bivalve native to the central Indo-Pacific, ranging from Myanmar to the Solomon Islands, including northern Australia and the South China Sea [1], is especially valued for its large, lustrous marine pearls. However, overfishing, climate change, and habitat degradation have led to the near disappearance of *P. maxima* populations in waters shallower than 15 m. This species is thus rare in the northern South China Sea [2], and this severely hindered the wild harvest of pearl oyster *P. maxima* as a sustainable source for pearl farming. Researchers have focused on germplasm resource analysis [3,4,5], artificial seed production and breeding [6,7,8,9,10]. However, high mortality in *P. maxima* farming, particularly at the 3–6 cm shell-height stage, has been attributed to diseases, environmental degradation, micro-algal composition, and other factors [11]. It has become a global concern.

Multi-omics integration indicated that pearl oyster *P. maxima* juveniles at peak mortality stages undergo oxidative stress, immune gene activation, and a worsening low-energy state [12]. Moreover, domestication offers significant opportunities for genetic enhancement [13]. Acclimatization studies of cultivated *P. maxima* have been previously conducted, both the F1 and F2 generations of the juvenile pearl oyster *P. maxima* (1 cm to 3 cm) experience significant mortality throughout the domestication process. However, preliminary results indicate that after two consecutive generations of acclimatization, the survival rate of cultured seedlings can increase by >10% [14]. Zhang et al. [15] identified multiple selection signatures, including genes involved in calcium signaling, immune defense, and metabolism (e.g., *PmCaM*, *PmCAP*, *PmHSP90*, *PmCLECT*, *PmTBC*, *PmASTL*, *PmJAG1*, and *PmPEX7*) during the domestication of pearl oyster *P. maxima*, which provide a foundation for our current transcriptomic and gut microbiota analysis of domesticated offspring. However, domestication has been a major challenge because of food availability, salinity, temperature, bacterial infections, and biofouling [12]. The gut, in particular, plays critical roles in digestion and nutrient assimilation [16,17], as well as pathogen resistance and immune modulation [18,19]. Recent advances in multi-omics integration have demonstrated that combined analysis of gut microbiota and host transcriptomics provides unparalleled insights into adaptive mechanisms [20,21].

However, integrated studies of the gut microbiota and transcriptomes in domesticated coastal *P. maxima* populations remain limited. Based on previous studies, we hypothesize that the high mortality observed in our domesticated *P. maxima* cohort stems from strong natural selection associated with rapid environmental changes [22]. Our goal was to identify markers of environmental adaptability in the domesticated population and provide scientifically grounded recommendations to guide future domestication strategies.

## 2. Materials and Methods

### 2.1. Experimental Materials and Sampling

In 2014, a total of 2000 pearl oysters *P. maxima* were collected from Sanya, Hainan, and farmed in Beibu Bay, Zhanjiang Guangdong. In 2018, we randomly selected 20 healthy, mature parent pearl oysters (F0) from the collected samples, with a 1:1 male-to-female ratio, and applied group breeding to produce the first domesticated generation (F1). After air-drying and inducing spawning through flow simulation, mature males and females released sperm and eggs, respectively. After fertilization, D-shaped larvae were collected at 48 h postfertilization and subsequently reared in tanks [7]. When eyed larvae appeared, plastic films were provided for settlement. Larval culture and rearing protocols were based on those described in a previous study Xie et al. [23]. On day 60, individuals measuring 2–3 mm in shell length were removed from plates, transferred to net cages, and suspended in Beibu Bay, Zhanjiang, China. Shells were cleaned and placed in new nets at appropriate intervals. The pearl oysters were regularly cleaned and relocated to new net cages to ensure optimal growth conditions.

To ensure the accuracy and rigor of the experimental results, standardized and precise sampling of *P. maxima* populations (F0 and F1) at identical growth stages (shell length: 13.43 ± 1.12 cm) from the same marine area across different time points was performed. At each sampling event, six individuals (three per group) were collected. All six samples passed quality control and were used for downstream analyses. After RNA extraction and quality assessment, three biological replicates with the highest integrity per group were selected for transcriptome sequencing and gut microbiota analysis; the remaining samples were excluded due to low RNA quality or outlier expression patterns. The intestinal tissue was carefully dissected from the edge of the adductor muscle using sterilized scissors and forceps. Gut contents were gently extracted using forceps and transferred into sterile 1.5 mL collection tubes, which were immediately and accurately labeled. Samples were promptly snap-frozen in liquid nitrogen and subsequently stored at −80 °C to preserve their integrity for downstream analyses.

### 2.2. High-Throughput Sequencing

#### 2.2.1. Transcriptome Sequencing (RNA-Seq)

Intestinal tissues from each individual (three per group) were separately cryogenically ground in liquid nitrogen, and total RNA was extracted individually using TRIzol reagent (Invitrogen™, Thermo Fisher Scientific, Waltham, MA, USA). A total of six RNA samples (three from F0 and three from F1) were submitted to Majorbio (Shanghai, China) for quality assessment and high-throughput sequencing. Quality control included quantification of RNA concentration and purity using a NanoDrop 2000 spectrophotometer (Thermo Fisher Scientific, Waltham, MA, USA), integrity evaluation via agarose gel electrophoresis, and determination of the RNA Quality Number (RQN) using an Agilent 5300 Bioanalyzer (Agilent Technologies, Santa Clara, CA, USA). For library construction, each RNA sample had to meet the following criteria: total RNA amount of ≥1 μg, RNA concentration ≥30 ng/μL, RQN > 6.5, and OD260/280 ratio between 1.8 and 2.2. mRNA was enriched using oligo(dT) magnetic beads, fragmented, and reverse-transcribed into double-stranded cDNA with random hexamer primers. After end-repair and blunt-end processing, Y-shaped adapters were ligated. The resulting constructs were purified, fragmented, size-selected, and PCR-amplified to generate the final sequencing libraries. Libraries were quantified using a Qubit 4.0 fluorometer, pooled, clustered via cBot bridge PCR, and sequenced on the Illumina NovaSeq X Plus platform at Majorbio, Shanghai, China.

#### 2.2.2. Data Quality Control, Transcriptome Assembly, and Differential Gene Expression Analysis

Raw transcriptomic data may contain sequencing adapters, low-quality reads, sequences with high N content, and excessively short fragments. Following quality control, clean reads were statistically analyzed and aligned to an unpublished *P. maxima* reference genome to obtain mapped reads for transcriptome assembly and gene expression quantification. Alignment metrics were assessed, and gene-level read counts were generated for identifying differentially expressed genes (DEGs) and conducting functional analyses. To ensure analytical robustness, raw sample data were further refined via the sequencing provider’s cloud platform. All six samples (three per group) passed quality control and exhibited high inter-sample correlation based on gene expression profiles. Therefore, all samples were included in the subsequent differential expression analysis and functional enrichment. Differential expression analysis was performed using DESeq2 (version 1.30.0; Bioconductor package; https://bioconductor.org/packages/DESeq2, accessed on 1 August 2026), with significance thresholds set at a false discovery rate padj (FDR) < 0.05 and |log_2_FC| ≥ 1. DEGs were annotated through BLASTx (https://www.ncbi.nlm.nih.gov/tools/primer-blast, accessed on 1 August 2026) searches against the GO and KEGG databases [24]. For the background dataset, all protein-coding genes annotated in the *P. maxima* reference genome were used as the universal background for both GO and KEGG enrichment analyses. GO enrichment analysis was conducted using Goatools and Fisher’s exact test. The FDR method was applied to adjust *p*-values for multiple testing, and GO terms with FDR < 0.05 were considered significantly enriched. KEGG pathway enrichment analysis was conducted using the Python SciPy package (version 1.10.0; https://scipy.org/, accessed on 1 August 2026) and the same statistical framework; the threshold for identifying significantly enriched pathways was a corrected *p*-value < 0.05.

#### 2.2.3. Gut Microbiota Detection

Total intestinal genomic DNA from the microbial communities was extracted separately from the intestinal microbiota of three *P. maxima* in the F0 and three in the F1. using the E.Z.N.A.^®^ Soil DNA Kit (Omega Bio-tek, Norcross, GA, USA) following the manufacturer’s protocol. DNA quality was evaluated by 1% agarose gel electrophoresis, and concentration and purity were assessed using a NanoDrop 2000 spectrophotometer (Thermo Fisher Scientific, Waltham, MA, USA). PCR amplification targeted the V3–V4 hypervariable regions of the 16S rRNA gene using the extracted DNA as the template. Amplification was performed with the primers 338F (5′-ACTCCTACGGGAGGCAGCAG-3′) and 806R (5′-GGACTACHVGGGTWTCTAAT-3′), which included unique barcode sequences [25]. The thermal cycling conditions were as follows: initial denaturation at 95 °C for 3 min; 27 cycles of denaturation at 95 °C for 30 s, annealing at 55 °C for 30 s, and extension at 72 °C for 30 s; and a final extension at 72 °C for 10 min. PCR reactions were conducted using a T100 Thermal Cycler (Bio-Rad Laboratories, Hercules, CA, USA).

Each 20 μL PCR reaction contained 4 μL of 5× TransStart FastPfu buffer, 2 μL of 2.5 mM dNTPs, 0.8 μL each of 5 μM forward and reverse primers, 0.4 μL of TransStart FastPfu DNA polymerase, 10 ng of template DNA, with the remainder made up with nuclease-free water. Amplification products were separated by 2% agarose gel electrophoresis and purified using a PCR Clean-Up Kit (Yuhua, Guangzhou, China). DNA concentrations were measured using a Qubit 4.0 fluorometer (Thermo Fisher Scientific, Waltham, MA, USA). Purified amplicons were used for library preparation with the NEXTFLEX Rapid DNA-Seq Kit, which involved (1) adapter ligation; (2) removal of adapter dimers using magnetic beads; (3) PCR enrichment of adapter-ligated fragments; and (4) final purification of amplified libraries with magnetic beads. Sequencing was performed on the Illumina PE300/PE250 platform at Majorbio (Shanghai, China).

#### 2.2.4. Statistical and Bioinformatics Analyses

Quality control of raw sequencing data from two groups (N = 3 per group) was performed using fastp [26] (version 0.19.6, https://github.com/OpenGene/fastp, accessed on 1 August 2026) and FLASH [27] (version 1.2.11, http://www.cbcb.umd.edu/software/flash, accessed on 1 August 2026). Low-quality bases (Q < 20) at the 3′ ends of reads were trimmed using a sliding window of 50 bp; if the average quality within the window fell below 20, bases from the start of the window were removed. Reads shorter than 50 bp or containing ambiguous bases (N) were discarded. Paired-end reads were merged into single sequences based on their overlap, requiring a minimum of 10 bp overlap and allowing a maximum mismatch rate of 0.2; reads not meeting these criteria were excluded. Samples were demultiplexed using barcodes and primers at both ends, allowing no barcode mismatches and up to two primer mismatches. Merged reads were then dereplicated, and singletons were removed.

Denoising of quality-filtered sequences was performed using the DADA2 plugin [28] within QIIME2 (version 2022.2) with the following parameters: --p-trim-left 0 --p-trunc-len 0 --p-trunc-q 0, which generated amplicon sequence variants (ASVs). All samples were rarefied to 35,597 sequences per sample for downstream diversity analyses. ASVs classified as chloroplast or mitochondria were removed. Taxonomic assignment of ASVs was carried out using the Naive Bayes classifier implemented in QIIME2, with the Silva 16S rRNA gene reference database (version 138). Functional potential of the gut microbial communities was predicted based on 16S rRNA gene data using PICRUSt2 [29].

### 2.3. Quantitative Real-Time PCR Validation (qRT-PCR)

Six intestinal RNA samples were used for qRT-PCR validation. Following the identification of DEGs, qRT-PCR validation was performed. Custom primers encompassing exon-exon junctions were formulated using Primer Premier 5 (Table 1), and primer specificity was confirmed by the NCBI Primer-BLAST tool (https://www.ncbi.nlm.nih.gov/tools/primer-blast, accessed on 1 August 2026) to guarantee unique amplification. cDNA was synthesized using the TransScript^®^ All-in-One First-Strand cDNA Synthesis SuperMix for PCR (TransGen Biotech, Beijing, China). qRT-PCR was carried out using PerfectStart^®^ Green PCR SuperMix (TransGen Biotech) following the manufacturer’s protocol. A melting curve analysis was conducted after amplification to confirm the specificity of the PCR products.

qRT-PCR reactions were run on a Roche LightCycler 480 system with a 10 μL reaction volume containing 5 μL of SYBR Green master mix, 0.4 μL of cDNA template, 0.4 μL each of forward and reverse primers, and 3.8 μL of double-distilled water [30]. *β-actin* was used as the internal reference gene [15], and relative gene expression levels were calculated using the 2^−ΔΔCt^ method with three technical replicates per sample.

## 3. Results

### 3.1. Transcriptome Analysis Between F0 and F1

**Figure 1:**
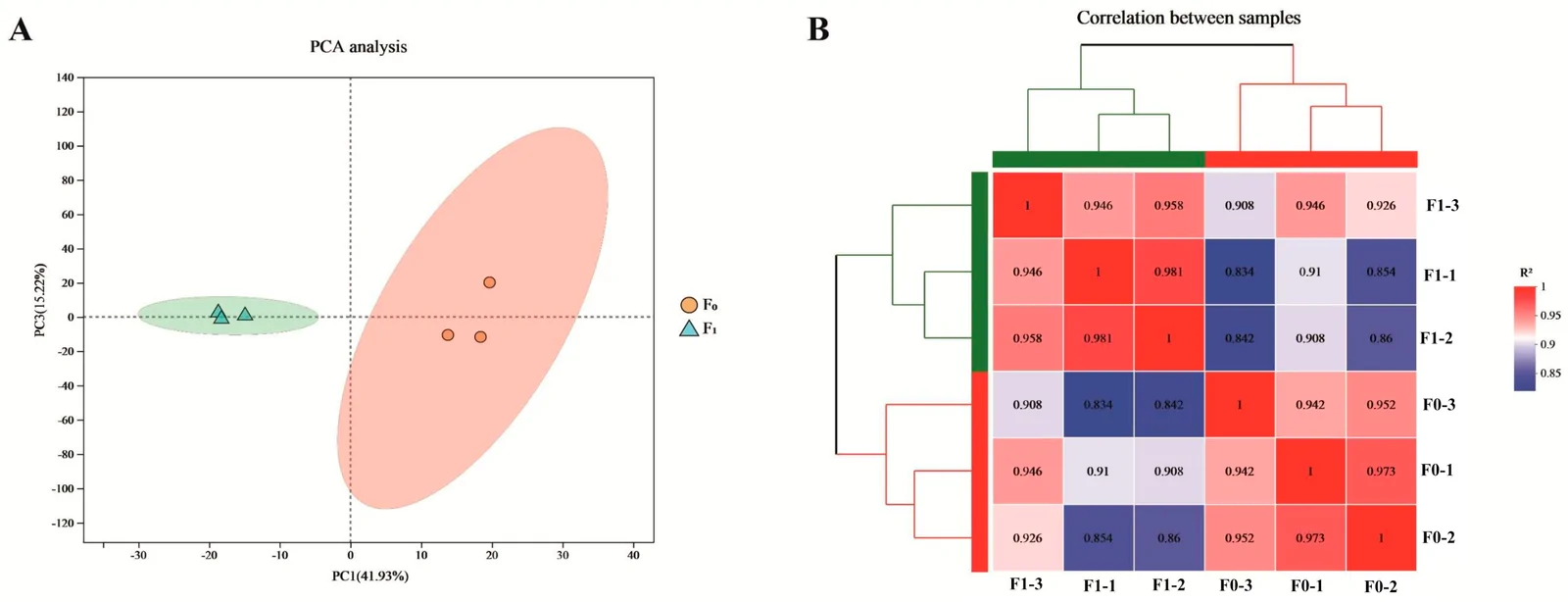
(**A**) PCA plot of intestinal transcriptomes from F0 (orange) and F1 (blue) groups of pearl oyster *P. maxima* (N = 3/group); percentages on axes indicate variance explained. (**B**) Pearson correlation heatmap of the same samples; heatmap color scale shows correlation coefficients (R). Analyses performed using DESeq2 in R.

A total of 264,094,684 raw reads were generated from the six transcriptome samples (three F0 and three F1 individuals). After quality control filtering, 261,603,152 high-quality clean reads were retained, with an average of 43,600,525 clean reads per sample. These reads exhibited high sequencing quality, with an average GC content of 39.37%, Q20 of 98.71%, and Q30 of 95.86%. All clean reads were aligned to the *P. maxima* reference genome using HISAT2 software (version 2.1.0; http://daehwankimlab.github.io/hisat2/, accessed on 1 August 2026), with mapping rates ranging from 63.91% to 79.48% across samples (Table 2). PCA based on similarity matrices revealed clear separation between the F0 and F1 groups (Figure 1A), and Pearson correlation analysis of FPKM values revealed strong consistency among biological replicates within each group (Figure 1B).

**Figure 2:**
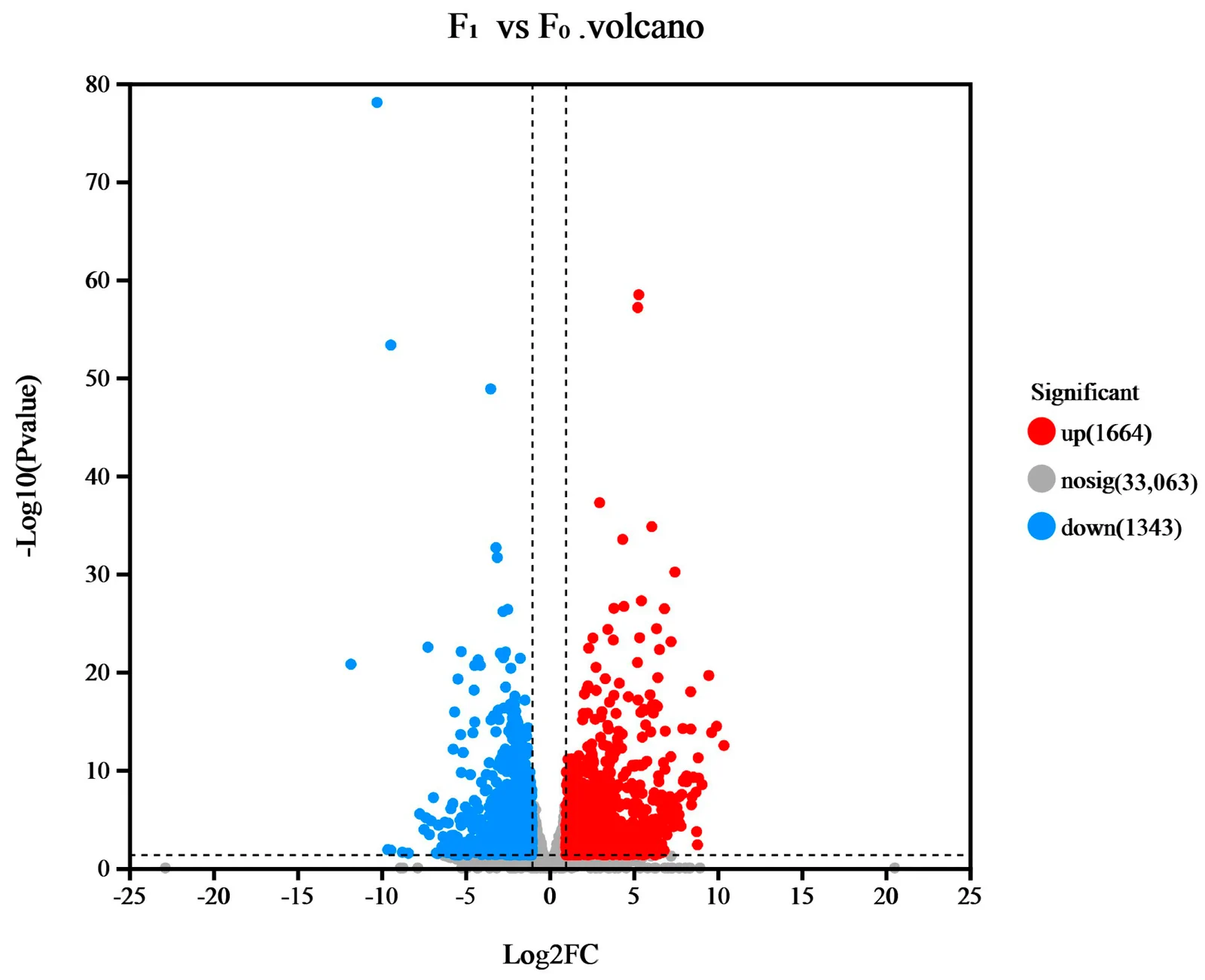
Volcano plot of DEGs between F1 and F0 groups of pearl oyster *P. maxima* (N = 3/group). Red: up-regulated; blue: down-regulated (|log_2_FC| ≥ 1, FDR < 0.05); gray: not significant. x-axis: log_2_FC; y-axis: −log_10_(FDR). DESeq2 with FDR correction.

DEGs identification utilized F0 as the control group, revealing a total of 3007 DEGs across the F1 and F0 groups, comprising 1664 up-regulated and 1343 down-regulated genes (Figure 2). These DEGs contained numerous genes involved in immune regulation, metabolism, and ion homeostasis.

**Figure 3:**
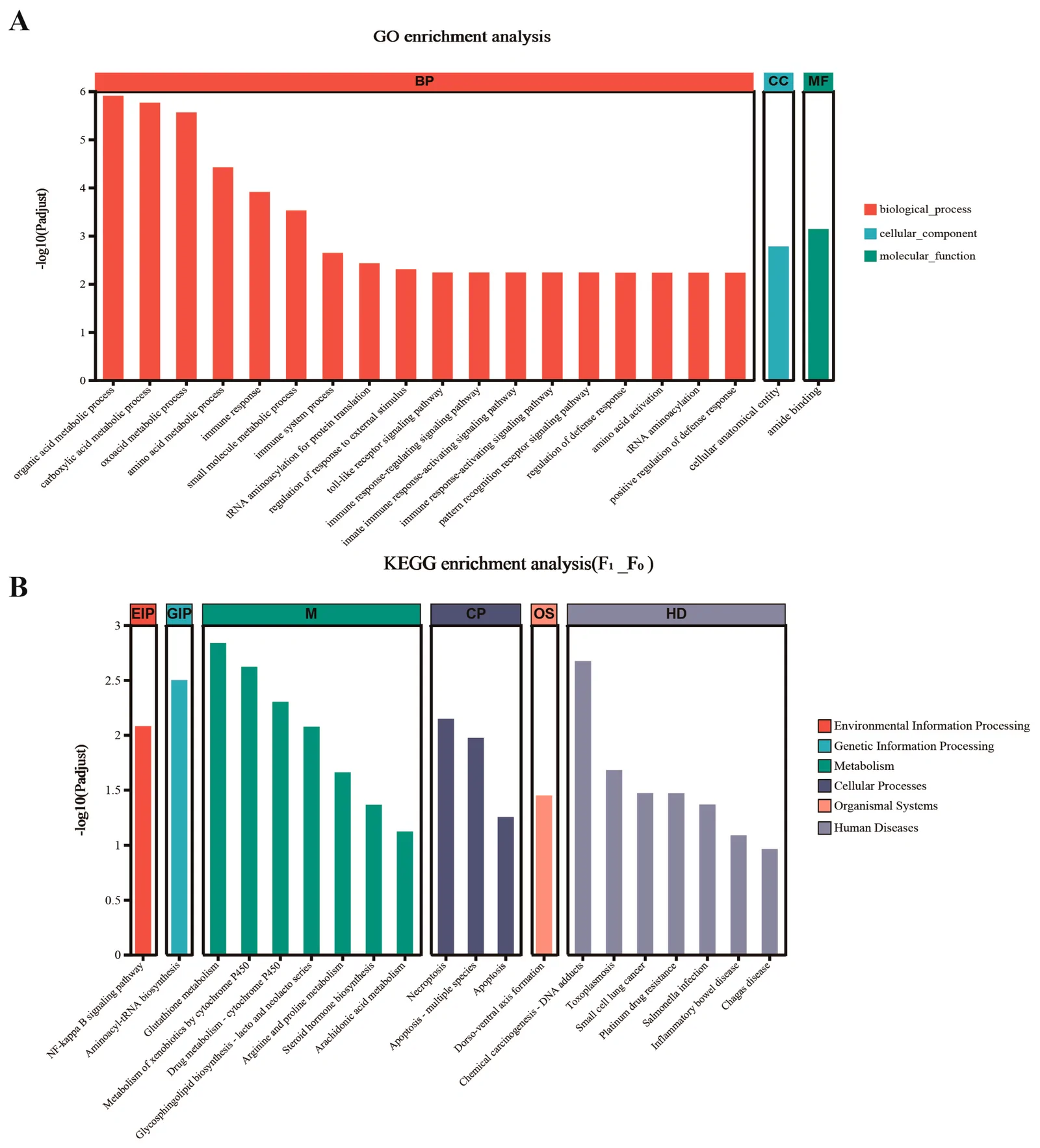
Functional enrichment of DEGs in pearl oyster *P. maxima* (N = 3/group). (**A**) GO classification (BP, CC, MF); (**B**) KEGG pathways. FDR < 0.05; bar colors indicate −log_10_(FDR); rich factor = enriched genes/total genes in pathway. Fisher’s exact test with FDR correction.

Functional enrichment analysis was performed on the 3007 DEGs, and the findings are detailed below. The 20 enriched GO terms in the F1 population were related to immunity, metabolism, and stress response. The most significantly enriched biological process terms included “organic acid metabolic process” (GO:0006082), “carboxylic acid metabolic process” (GO:0019752), and “oxoacid metabolic process” (GO:0043436). “Cellular anatomical entity” and “amide binding” were enriched in the cellular component and molecular function categories, respectively (Figure 3A). In metabolic pathways, DEGs were significantly enriched in “glycosphingolipid biosynthesis—lacto and neolacto series” (KO00603) and “metabolism of xenobiotics by cytochrome P450” (KO00980). Pathways related to immunity and signal transduction included “NF-kappa B signaling pathway” (KO04064), “inflammatory bowel disease” (KO05321), and “Salmonella infection” (KO05132) (Figure 3B).

### 3.2. Analysis of Differences in the Gut Microbiota of Domesticated Offspring

#### 3.2.1. Differences in the Composition and Diversity of the Gut Microbiota Community

High-throughput 16S rRNA sequencing was performed to investigate the effect of domestication on the composition of the gut microbiota. A total of 601,880 clean reads were obtained from intestinal samples (six samples in total), with an average of 100,313 sequences per sample after rarefaction. A total of 2293 ASVs were identified.

**Figure 4:**
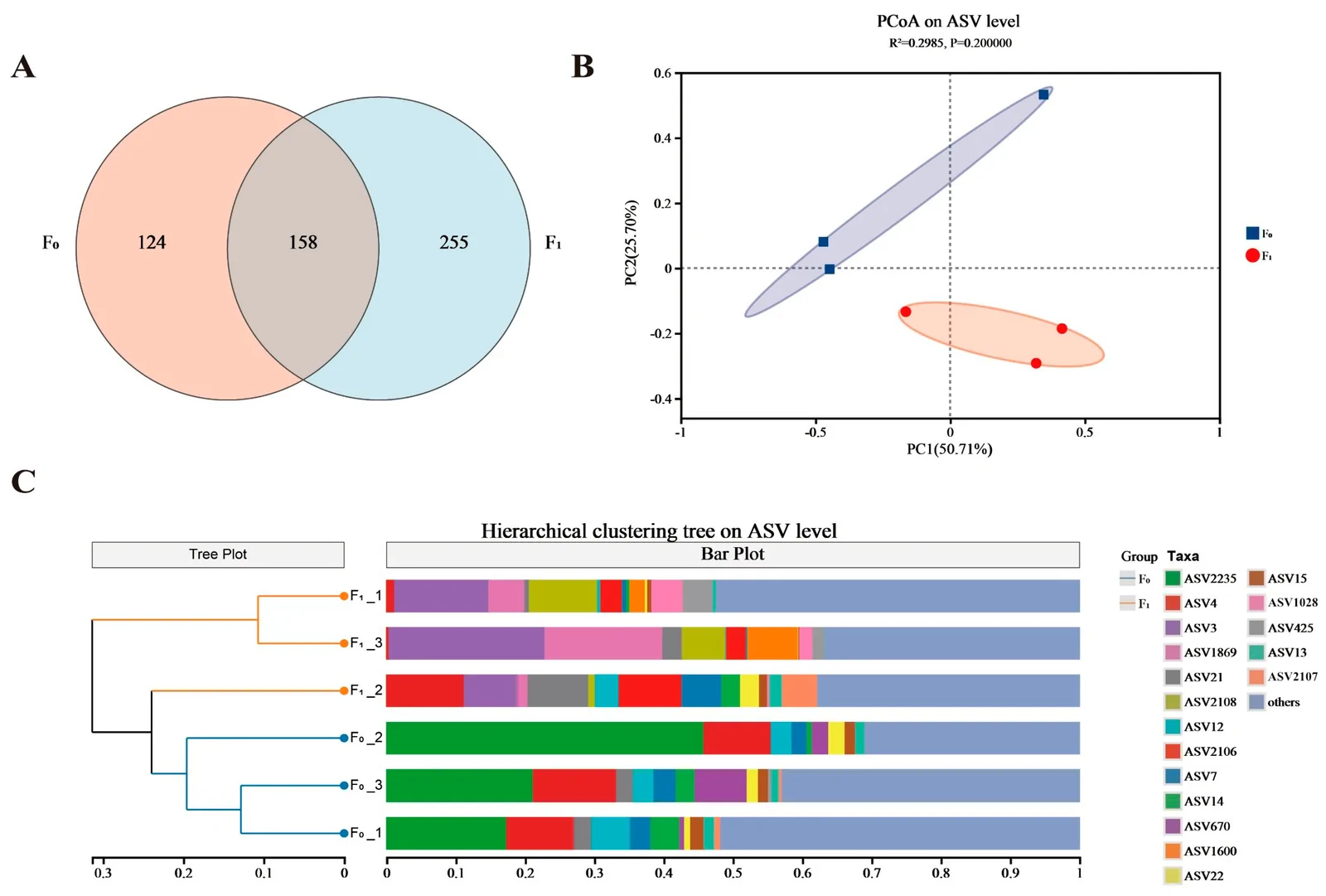
Analysis of the relationship among gut microbiota samples of pearl oyster *P. maxima* (N = 3/group). (**A**) Venn diagram of gut microbiota ASVs in F0 and F1 groups. (**B**) Principal coordinate analysis (PCoA) of F0 and F1 groups. (**C**) Hierarchical clustering of samples in F0 and F1 groups.

Compared with F0 individuals, F1 individuals exhibited higher ACE, Chao1, Shannon, and Sobs diversity indices but a lower Simpson index. Although these differences in richness and diversity metrics were not statistically significant (*p* > 0.05) (Table 3). Analysis of the Venn diagram at the genus level identified 158 shared ASVs, with 124 unique ASVs in the F0 group and 255 unique ASVs in the F1 group (Figure 4A). Principal coordinate analysis (PCoA) at the ASV level exhibited a visible separation between F0 and F1 samples, with PC1 and PC2 explaining 50.71% and 25.7% of the variance, respectively, suggesting a potential shift in the intestinal microbial community structure (Figure 4B). Phylogenetic tree analysis further supported this distinction; all samples were clustered into two separate branches corresponding to F0 and F1. F1 samples characterized by a high abundance of ASV3 were closely clustered, and ASV2235 was relatively abundant in F0 but rare or absent in F1 (Figure 4C).

#### 3.2.2. Differences in Specific Microbial Taxa

**Figure 5:**
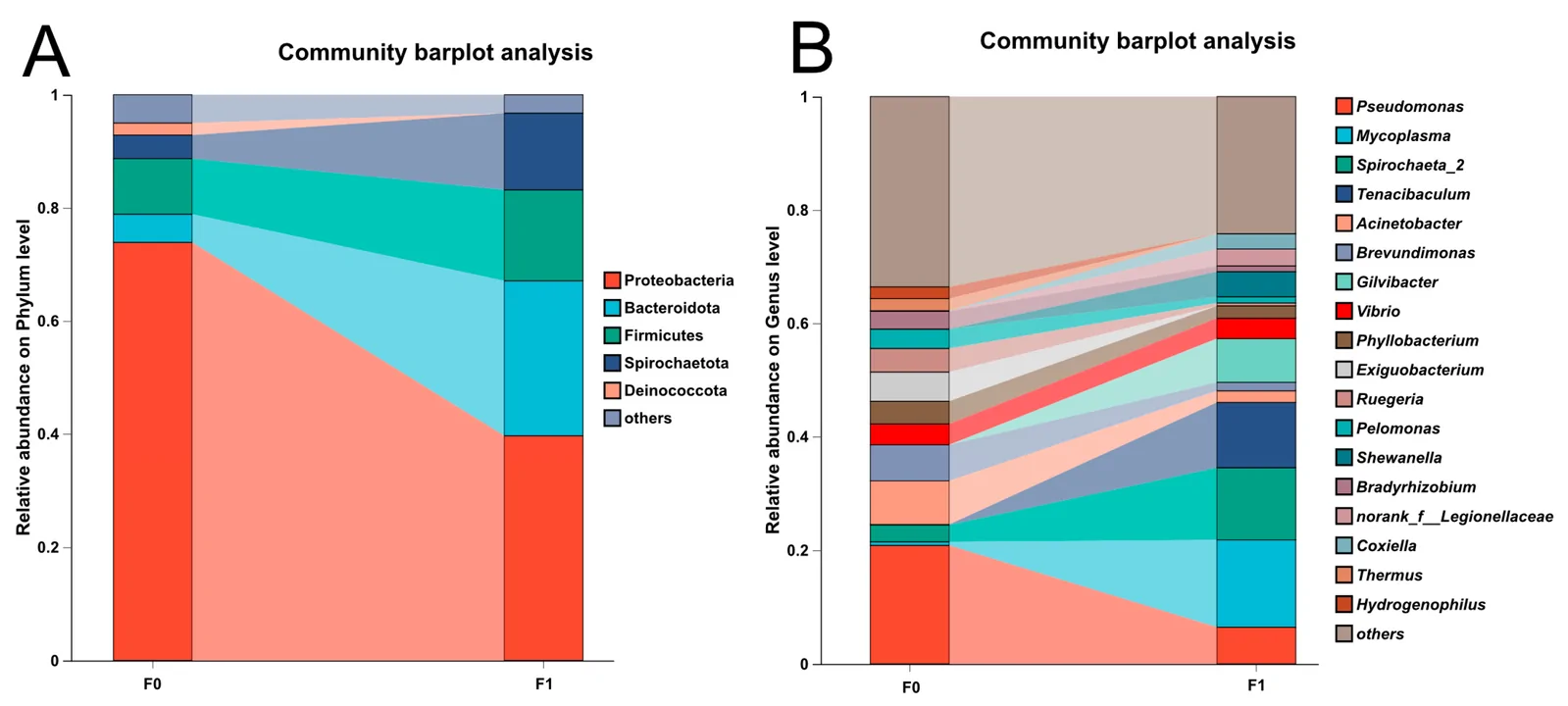
Analysis of the differential distribution of gut microbiota in domestication groups of pearl oyster *P. maxima* (N = 3/group). (**A**) Relative abundance of microbial communities at the phylum level. (**B**) Relative abundance of microbial communities at the genus level.

Changes in the relative abundance of dominant taxa were observed at both the phylum and genus levels. At the phylum level, the F0 gut microbiota primarily comprised Proteobacteria, Firmicutes, and Cyanobacteria, whereas the F1 microbiota was dominated by Proteobacteria, Spirochaetota, Bacteroidota, and Cyanobacteria; Proteobacteria was the most prevalent in both groups. At the genus level, F0 samples were predominantly enriched in *Mycoplasma*, *Pseudomonas*, while F1 samples were dominated by *Spirochaeta_2*, *Mycoplasma*, and *Pseudomonas* (Figure 5B).

#### 3.2.3. Functional Prediction of Gut Microbiota

**Figure 6:**
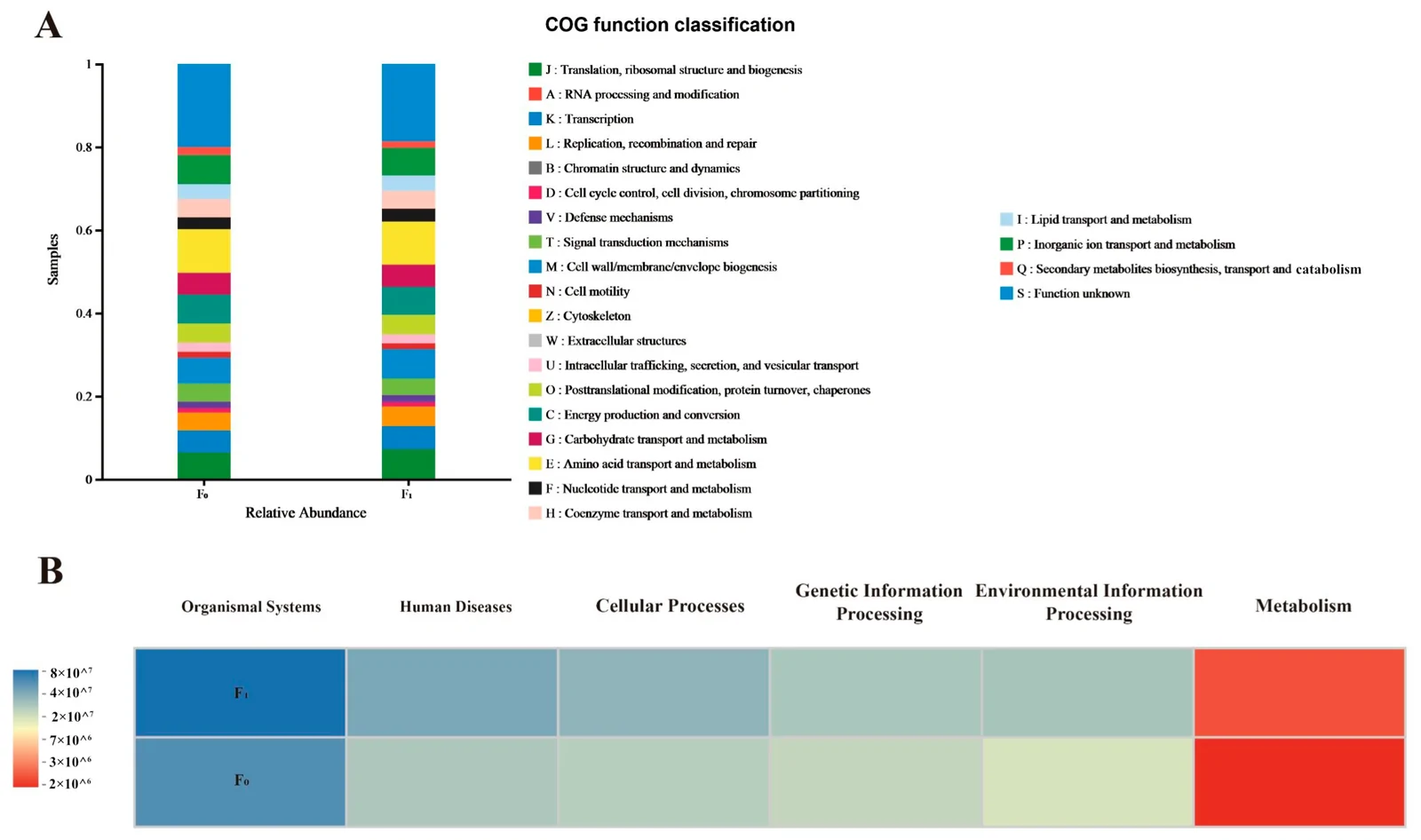
PICRUSt2 functional prediction of gut microbiota in pearl oyster *P. maxima* (N = 3/group). (**A**) Differences in COG (Clusters of Orthologous Groups) functional abundance. (**B**) KEGG (Kyoto Encyclopedia of Genes and Genomes) pathway enrichment at Level 1.

The predicted COG and KEGG functional profiles of microbial orthologous groups indicated disparities in the amount of functional genes between the F1 and F0 groups. The disparities were particularly evident in pathways associated with metabolism, immunological response, apoptosis, and cell signalling, aligning with the results of the transcriptome analysis, indicating a possible gene–microbial functional synergy mechanism. The enriched functions of the gut microbiota of F1 were associated with translation, ribosomal structure, and biogenesis. Additionally, pathways related to metabolism and environmental information processing were significantly enriched (Figure 6).

### 3.3. Validation of RNA-Seq Data by qRT-PCR

**Figure 7:**
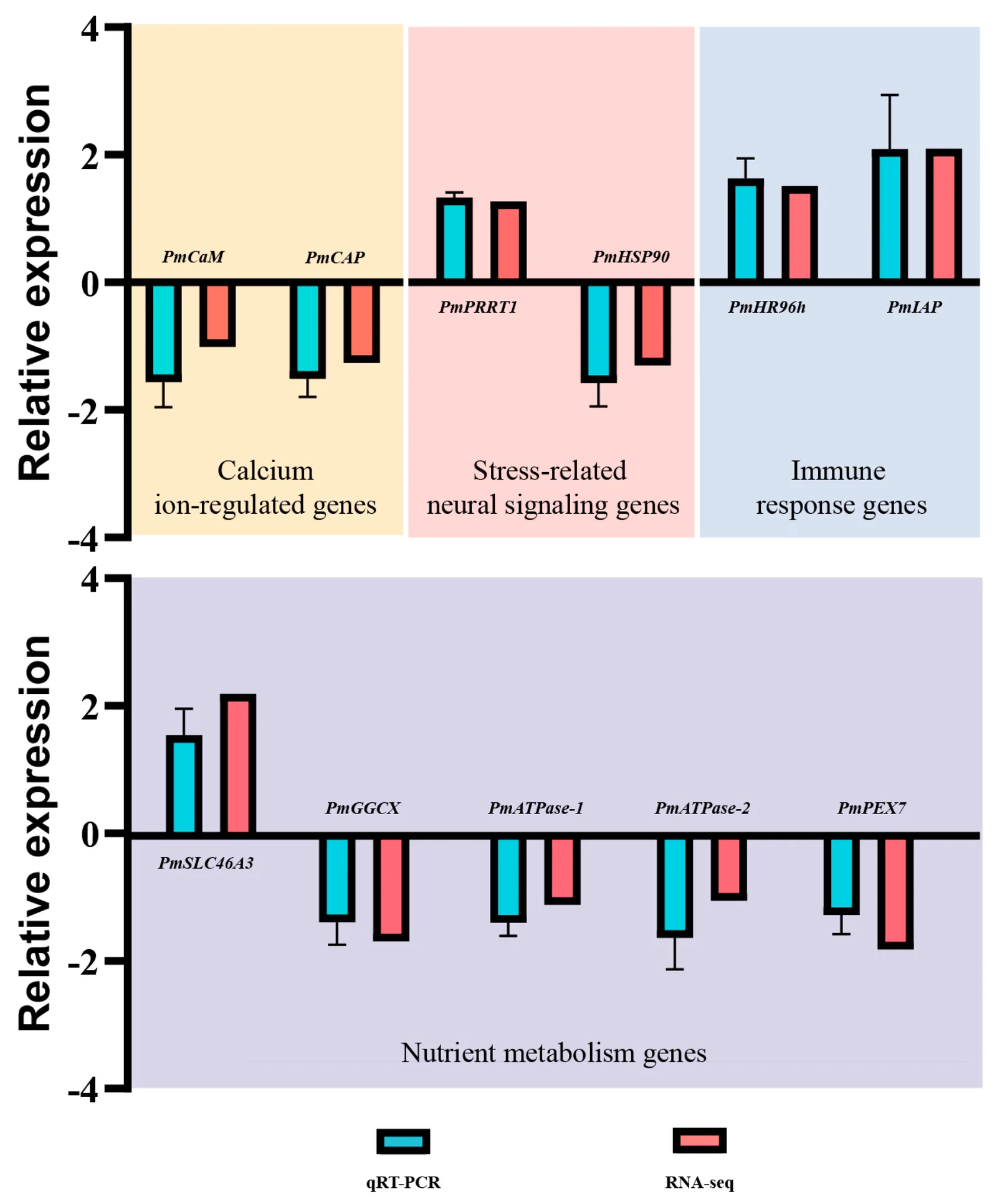
qRT-PCR validation of 11 DEGs in F1 vs. F0 groups of pearl oyster *P. maxima* (N = 3/group). Bars represent mean fold change (2^−ΔΔCt^) ± SD. *β-actin* was used as the internal control. Statistical analyses were performed using independent *t*-tests.

To corroborate the intestinal transcriptome findings, qRT-PCR was used to assess the expression levels of 11 differentially expressed genes, classified by their predominant enriched functional pathways (Figure 7). These genes were grouped into four functional categories based on their predominant enriched pathways: immune response (*PmHR96h*, *PmIAP*), nutritional metabolism (*PmSLC46A3*, *PmPEX7*, *PmATPase-1*, *PmATPase-2*, *PmGGCX*), neural signaling (*PmPRRT1*, *PmCAP*), and calcium ion regulation (*PmCaM*, *PmHSP90*). Among the immune-related genes, *PmHR96h* and *PmIAP* were significantly up-regulated in F1 compared with F0 (mean fold change ± SD: 2.35 ± 0.32 for PmHR96h, *p* < 0.01; 1.89 ± 0.21 for PmIAP, *p* < 0.05). Within the metabolism-related group, *PmSLC46A3* was significantly up-regulated (fold change: 2.12 ± 0.28, *p* < 0.01). Of the genes involved in signaling pathways, *PmCAP*, *PmCaM*, and *PmHSP90* were significantly down-regulated (fold changes: 0.52 ± 0.06, 0.48 ± 0.05, and 0.38 ± 0.04, respectively; all *p* < 0.01), while *PmPRRT1* was notably up-regulated (fold change: 1.78 ± 0.22, *p* < 0.05). Moreover, the qRT-PCR data were consistent with the RNA-seq results from the F1 group, further confirming the reliability of the transcriptomic data.

## 4. Discussion

This study employed integrated transcriptomic and gut microbiota profiling to assess the influence of nearshore domestication on *P. maxima* offspring. Transcriptomic analyses showed significant upregulation of immune-related genes and altered expression of genes involved in metabolism and neuro-signaling, suggesting physiological acclimatization to coastal stressors.

### 4.1. Transcriptomics Reveals the DEGs in Domesticated Offspring

Transcriptomic analyses have proven to be effective in identifying genes and elucidating the pathways underlying responses to environmental changes, and they have been widely used to explore the potential trade-offs associated with domestication [31]. Comparative transcriptomic studies in domesticated animals are commonly employed to investigate biological processes related to immune and stress responses [32], digestive function [33], and the metabolism of various compounds [34]. In *P. maxima*, previous studies have documented the effects of salinity and temperature stress on thermal tolerance [35] and immune function [36]. Environmental stressors are known to induce significant changes in gene expression in the intestines of bivalves. Our findings reveal consistent transcriptomic alterations in domesticated *P. maxima*, particularly in pathways related to immunity, metabolism, and neuro-signaling, likely reflecting acclimation to nearshore farming conditions.

#### 4.1.1. Neural Signal Transmission

An organism’s adaptability is closely tied to its ability to respond to both internal and external stimuli, and rapid molecular responses to environmental changes are essential for survival. These responses depend on cellular mechanisms that mediate the perception, transmission, storage, and interpretation of signals received through various pathways [37]. Similar to other vertebrates, shellfish exhibit stress responses when exposed to adverse environmental conditions. Distinct within-group clustering was observed among experimental groups, and 3007 DEGs were identified in the intestinal tissues of domesticated *P. maxima* offspring.

Significant changes in the expression of *HSP90*, which is annotated as a stress-inducible molecular chaperone based on sequence homology, were observed in the F1 generation. While *HSP90* has been characterized as a stress-response protein in other organisms, its specific function in *P. maxima* remains to be experimentally validated. Previous studies in C. *gigas* have shown that exposure to paralytic shellfish toxins disrupts ion-channel and neuromuscular pathways [38], suggesting that similar pathways may be affected in *P. maxima* by nearshore environmental changes such as increased acidity and pollution, which may alter intracellular calcium influx and downstream Ca^2+^-dependent metabolism [39]. Calmodulin (*PmCaM*) is predicted to bind calcium based on sequence homology and has been reported to mediate myosin–actin interactions in smooth muscle contraction in other taxa [40], leading us to hypothesize that it may play a role in neural signaling and digestion in pearl oyster *P. maxima*. Similarly, the functional interpretations of *PmCAP*, *PmPRRT1*, and other ion-regulated genes are based on GO/KEGG annotations and homology to genes characterized in vertebrates and model organisms [41,42,43,44]. Therefore, we interpret the observed down-regulation of *PmCaM* and *PmCAP* in domesticated offspring as suggestive of correlative shifts in calcium-related signaling in *P. maxima*.

#### 4.1.2. Analysis of Differences in the Vitamin Absorption Capacity

Protein post-translational modifications play a critical role in cellular signal transduction, and phosphorylation is one of the most essential and ubiquitous regulatory mechanisms in biological systems. Substantial changes in protein phosphorylation were observed during the domestication of *P. maxima*. The expression of NADH dehydrogenase subunit *NDUFB7* and V-type H^+^-transporting ATPase subunits was markedly elevated during oxidative phosphorylation [45]. These enzymes are vital for proton gradient formation and ATP synthesis, suggesting that the increased expression of these genes enhanced ATP production and energy transfer to facilitate the adaptation of oysters to nearshore conditions.

Furthermore, phosphorylation-related genes may influence neuronal transmission. *SLC46A3*, a transporter located on the apical membrane of small intestinal epithelial cells, mediates the transmembrane transport of branched-chain amino acids (BCAAs: leucine, isoleucine, and valine) from the intestinal lumen into epithelial cells. It may also affect the absorption of other nutrients and the bioavailability of vitamins and metabolites. BCAAs are important energy sources and participate in metabolic pathways, including glutamine synthesis. Glutamine acts as a cofactor in the metabolism of several vitamins, such as folic acid and niacin. Previous studies have shown that *SLC46A3* mutant mice exhibit significantly elevated hepatic copper levels, indicating that *SLC46A3* might play a role in copper transport into lysosomes [46].

Another gene, *PEX7*, is involved in lipid metabolism. Mutations in *PEX7* in *Drosophila* are associated with increased circulating non-esterified fatty acids, which indicates that lipid metabolism is impaired [47]. Our findings suggest that environmental factors associated with nearshore domestication influence the expression of genes involved in amino acid and fatty acid metabolism, which affects the metabolic state of the domesticated offspring.

The *GGCX* gene encodes an enzyme responsible for γ-carboxylating specific glutamate residues in vitamin K-dependent proteins, which is essential for coagulation, bone metabolism, vascular calcification, and cell proliferation in host–microbiome metabolism [48]. In bivalves, lysosomes play a central role in immune defense by degrading foreign substances and necrotic cells through lysosomal enzymes, reactive oxygen species, nitric oxide, and other antimicrobial compounds [49]. The elevated expression levels of *PmSLC46A3* and *PmGGCX* observed in the transcriptome of domesticated progeny may reflect a negative feedback mechanism in response to changes in intestinal ion homeostasis and metabolism-related genes (*PmATPase*, *PmPEX7*), which helps maintain intracellular energy balance under domesticated conditions.

#### 4.1.3. Analysis of Differences in Immune Adaptation and Oxidative Stress

Although bivalves lack vertebrate-like adaptive immune cells and antibodies, they show marked immunological adaptability primarily through the expression of immune-related genes. Under low-temperature conditions, for example, bivalves can enhance their immune defenses by regulating the expression of these genes [44]. Their humoral immunity relies on a diverse array of enzymes and peptides secreted by immune cells, and apoptosis plays a key role in their defense mechanisms.

Bivalve humoral immune responses involve enzymes such as alkaline phosphatase and lysozyme, which possess antibacterial properties and enhance phagocytosis; lectins that recognize and bind to pathogen-associated carbohydrates, facilitating aggregation and clearance; antimicrobial peptides that directly eliminate pathogens; and reactive molecules including nitric oxide, superoxide anions, and protease inhibitors. Bivalves possess a diverse array of innate immune receptors, such as Toll-like receptors, RIG-I-like receptors, and C-type lectins, far exceeding the number found in humans and other model organisms [50].

Inhibitor of apoptosis (IAP) family proteins function by binding to the N-terminal IBM (IAP-binding motif) of activated caspases through their BIR (baculovirus IAP repeat) domain, thereby preventing caspase–substrate interactions and inhibiting apoptotic activity. Heat shock proteins (HSPs) are also critical to the stress response and facilitate the refolding of denatured or newly synthesized proteins during periods of cellular stress [51]. In most organisms, HSPs can constitute 5–10% of total cellular proteins under abiotic stress conditions.

The *HR96h* gene encodes gp96, a member of the HSP90 family and one of the most abundant proteins in the endoplasmic reticulum, where it functions as a molecular chaperone. Expansion of immune-related gene families in bivalves promotes structural and functional diversification, which allows these genes to take on novel roles in responses to environmental stressors [52]. In *Crassostrea* gigas, more than half of the identified immune-related receptors and adaptor proteins are associated with abiotic stress responses [50].

In this study, transcriptomic analysis of domesticated *P. maxima* offspring revealed the elevated expression of key immune-related genes, including *PmHR96h* and *PmIAP*, suggesting that immune pathways are activated in response to nearshore environmental conditions. These findings reveal an adaptive immunological response in domesticated *P. maxima*, which reflects enhanced resilience to coastal stressors.

### 4.2. Differences in the Gut Microbiota of Domesticated Offspring

Human activities have a profound influence on evolutionary processes and affect speciation through domestication, hunting, and ecosystem engineering [53]. Domestication in particular involves the intentional modification of animal nutrition, behavior, habitat, and reproduction by humans [54]. Through these interventions, anthropogenic forces have not only altered the genetic makeup and phenotypic traits of domesticated species but may have also reshaped their gut microbiota [55]. The gut microbiome plays a vital role in host health and immune function in humans [56], and it has been shown to have similar effects in terrestrial animals [57] and aquatic species [58,59]. We examined the effect of human-driven domestication and environmental changes in aquaculture on gut microbial communities by comparing the intestinal microbiota of F1 (domesticated) and F0 (wild-type) *P. maxima*.

Domestication plays a key role in facilitating the adaptation of bivalves to novel environmental conditions, and the gut microbiota significantly contributes to this process. Previous research has shown that the diversity and stability of gut microbial communities underlie the responses of bivalves to environmental changes. Under extreme conditions, such as low temperatures, the composition of the gut microbiota undergoes substantial shifts, often resulting in an increased abundance of beneficial bacteria that enhance the host’s stress resistance. The gastrointestinal microbiota are vital for bivalve growth and survival [60,61], aid digestion and energy regulation, inhibit pathogen colonization, and support mucosal immune function [61,62].

In both F0 and F1 *P. maxima* populations, the predominant intestinal bacterial phyla included *Proteobacteria*, *Firmicutes*, *Cyanobacteria*, *Spirochaetota*, and *Bacteroidota*. *Bacteroidota* are adept at degrading complex carbohydrates such as cellulose and hemicellulose into absorbable small molecular sugars, thereby supporting host energy metabolism, suggesting that the metabolic efficiency in F1 individuals maybe enhanced. At the genus level, shifts were observed in the dominant taxa of the domesticated group. These shifts in microbial composition suggest that gut microbiota were shaped by selective pressures imposed by the farming environment during the domestication of *P. maxima*.

### 4.3. Integrated Summary and Implications

In summary, this study reveals coordinated transcriptional and microbial shifts in domesticated *P. maxima*. Transcriptomic analysis showed changes in genes involved in immune defense, nutrient metabolism, and neural signaling, while 16S rRNA sequencing revealed restructuring of gut microbiota. Although causal relationships cannot be established, these findings suggest a coordinated host-microbiota acclimation to nearshore conditions. The identified candidate genes and bacterial biomarkers provide a foundation for future mechanistic studies and may inform selective breeding strategies. Limitations include small sample size and the correlative nature of the data, warranting further functional validation.

## 5. Conclusions

This study demonstrates that nearshore domestication reshapes both the intestinal transcriptome and gut microbiota of *P. maxima* offspring. Transcriptomic analysis revealed 3007 differentially expressed genes, with significant upregulation of immune-related genes and altered expression of metabolic and neural-signaling pathways, indicating physiological acclimatisation to coastal stressors. Concurrent 16S rRNA sequencing uncovered shifts in microbial community composition and increased alpha diversity in F1 individuals, while functional predictions pointed to enhanced metabolic and immune capacities. These coordinated host–microbiome changes reflect a multifaceted adaptive response, providing valuable molecular markers for selective breeding and health management in pearl oyster aquaculture under changing environmental conditions.

## Figures and Tables

**Table 1:** Primer sequences for qRT-PCR validation of selected DEGs. *β-actin* used as internal reference. Gene IDs from *P. maxima* reference genome.

Premier	Sequence (5′-3′)	Gene ID	PCR Efficiency (%)
*βactin*-F	CGGTACCACCATGTTCTCAG	-	-
*βactin*-R	GACCGGATTCATCGTATTCC		
*PmCaM*-F	ACATCTTCACCCTCTACTCCCTT	PINMA_Chr08_0789	93.5
*PmCaM*-R	GTTCTTCTTTGGTTATAGTTCCGTC		
*PmCAP*-F	CTTCGTGACCCTAAAACAGGC	PINMA_Chr03_1114	95.7
*PmCAP*-R	CTAACTCCCCAAAACTCATTCCT		
*PmPRRT1*-F	CCACATTTTTCTGTTTCTTCCC	PINMA_UnChr485_0001	95.4
*PmPRRT1*-R	ACTTGATTTTGCTCTTTCCACG		
*PmHSP90*-F	AAAAGGTGGTGGTATCCAACAGG	PINMA_Chr08_1169	92.8
*PmHSP90*-R	TGATGGAATGGTCAGGGTTTACTT		
*PmSLC46A3*-F	AATGGAGCAGAAGATTCAAGAGG	PINMA_Chr05_1400	96.4
*PmSLC46A3*-R	AAGCGGTATCAGGAGGCAA		
*PmGGCX*-F	ATCAAGTCAGATGAAAAGACCAAAA	PINMA_Chr09_0172	92.5
*PmGGCX*-R	GGGAGAACGGCAGGAAAGA		
*PmATPase*-1F	GCTTACCGGTTCGATTCTGC	PINMA_Chr01_1040	93.8
*PmATPase*-1R	CATCTGCTTTGTGTTCTCTTGTTG		
*PmATPase*-2F	GGCTAGATTCTATGCCCCATC	PINMA_Chr12_1494	95.7
*PmATPase*-2R	CTGTTTTCATTCTCCTACTCCCTT		
*PmPEX7*-F	AGCCTCAAGCTCATATGATTTCAC	PINMA_Chr12_2677	94.4
*PmPEX7*-R	CACAGTCCGCCATTTCTCC		
*PmHR96h*-F	TTATGATGTTGAAAATGATGGCTG	PINMA_Chr10_1478	92.5
*PmHR96h*-R	AGTACACTGTTGTTCTCACGGGT45		
*PmIAP*-F	AATACGAAAACGTCACAGCCC	PINMA_Chr12_1989	93.8
*PmIAP*-R	AACAGTAGCATCTTACCAAGTCCA		

**Table 2:** Summary of sequencing quality and annotation results for the transcriptomic analysis of F0 and F1 pearl oyster *P. maxima*.

Sample	Raw Data	Clean Data	Q20%	Q30%	GC Content%	Mapped Reads
F0-1	43,120,106	42,734,698	98.81%	96.18%	39.66%	27,311,262 (63.91%)
F0-2	41,575,864	41,214,026	98.84%	96.25%	38.94%	28,093,623 (68.17%)
F0-3	41,444,082	41,092,914	98.84%	96.27%	39.02%	28,532,055 (69.43%)
F1-1	44,915,198	44,416,724	98.29%	94.31%	39.72%	32,742,705 (73.72%)
F1-2	47,382,150	46,916,138	98.81%	96.19%	39.48%	34,716,735 (74.0%)
F1-3	45,657,284	45,228,652	98.72%	95.94%	39.3%	35,946,571 (79.48%)

**Table 3:** Alpha-diversity indices of gut microbiota in pearl oyster *P. maxima* (F0 vs. F1, N = 3/group). Values: mean ± SD. *p*-values from independent *t*-tests; all *p* > 0.05 (not significant).

Parameter	F0	F1	*p*-Value
ACE	175.96 ± 18.34	283.98 ± 42.67	0.107
Chao	174.91 ± 18.02	283.47 ± 42.33	0.106
Shannon	2.84 ± 0.41	3.22 ± 0.35	0.642
Simpson	0.27 ± 0.09	0.15 ± 0.06	0.487
Coverage	0.999 ± 0.001	0.999 ± 0.001	0.853
Sobs	173.80 ± 17.89	282.80 ± 42.15	0.104

Note: Values are presented as mean ± standard deviation (SD) for each group (N = 3). *p*-values were calculated using independent *t*-tests.

## Data Availability

The raw sequence data have been deposited in the Genome Sequence Archive (Genomics, Proteomics & Bioinformatics, 2025) at the National Genomics Data Center (Nucleic Acids Res, 2026), China National Center for Bioinformation/Beijing Institute of Genomics, Chinese Academy of Sciences, with accession numbers GSA: CRA047329 (16S rRNA gut microbiota data) and GSA: CRA047335 (transcriptome data), and are publicly available at https://ngdc.cncb.ac.cn/gsa, accessed on 1 August 2026.
